# Reflux gastro œsophagiens du prématuré: à propos d'un cas

**DOI:** 10.11604/pamj.2016.25.243.10497

**Published:** 2016-12-20

**Authors:** Daouda Ndour

**Affiliations:** 1 Service de Néonatologie et Réanimation Néonatale, Hôpital d'Enfants Albert Royer de Dakar, Université Cheikh Anta Diop de Dakar, Sénégal

**Keywords:** Premature infant, Gastroesophageal reflux, apnea, Premature infant, gastroesophageal reflux

## Abstract

Le reflux gastro-œsophagien (RGO) est un problème fréquent en néonatologie. Le RGO est souvent incriminé dans diverses manifestations cliniques survenant chez le prématuré. Diverses réponses reflexes physiologiques fournissent un lien biologique possible entre le reflux gastro-œsophagien; l'apnée et les bradycardies du prématuré sans que lien de causalité ne soit clairement établi. En outre les explorations et le traitement proposés chez le prématuré présentant un reflux sont très discutés. Nous rapportons le cas d'un nouveau-né prématuré admis en soins intensifs néonatals et présentant des épisodes d'apnées et de bradycardies. L'examen clinique et les résultats des explorations étaient normaux. Le diagnostic de reflux gastro œsophagien compliqué d'apnée et de bradycardie a été retenu. Le diagnostic de RGO est difficile à confirmer malgré l'arsenal des examens complémentaires. Le traitement médicamenteux n'a pas montré de réel intérêt pour l'amélioration des symptômes et devrait être réservé aux formes sévères. Nous nous proposons à partir de ce cas clinique de faire une revue de la littérature pour une mise au point sur les différents aspects du problème.

## Abstract (en)

Gastroesophageal reflux disease (GERD) is a common problem in neonatology. Various physiological protective reflex responses provide a plausible biological link between gastro-esophageal reflux and apnea and bradycardia in premature. It is uncertain whether or not there is a causal relationship between the two diseases. However there is no consensus about the clinical and paraclinical diagnosis. Further explorations and treatment offered to premature infants with symptoms are discussed. We report the case of a preterm infants admitted to neonatal intensive care and with apnea and bradycardia. Clinical examination and exploration results were normal. We retained the gastrointestinal reflux diagnosis complicated of apnea and bradycardia. Pharmacological therapy for gastro-esophageal reflux disease has not definitively been shown to be effective in improving symptoms and should be reserved especially for infants with treatment refractory apnea and bradycardia episodes suspected as being gastro-esophageal reflux in premature infants. From a case report we made a literature review to discuss at length the different aspects of the problem.

## Introduction

Le reflux gastro-œsophagien (RGO) est définit comme le passage intermittent et involontaire d'une partie du contenu gastrique dans l'œsophage. Il peut être associé à des régurgitations. Il s'agit d'un problème assez fréquent en néonatologie [1]. Le reflux gastro œsophagien fait partie des problèmes majeurs rencontrés dans les premiers jours de vie des nouveaux nés prématurés au même titre que les difficultés de régulation thermique, d´acquisition des compétences d´alimentation par voie orale, ainsi quele problème de l´apnée et l´établissement d'un rythme respiratoire normal. C'est donc une étape majeure du développement pour de nombreux prématurés. Le RGO peut se manifester surtout dans les premiers jours de vie par des épisodes d'apnées et de bradycardie. L'apnée du prématuré est définie comme une pause respiratoire de plus de 20 s, et/ ou accompagnée de désaturation en oxygène [saturation d´oxygène (SpO_2_) ≤ 80% pour ≥ 4s] et debradycardie (Fréquence cardiaque <2 /3 de la fréquence de référence pendant un temps ≥ 4s), chez les nouveaux nés de moins de 37 semaines de gestation. Le RGO est surtout favorisé par des facteurs liés à la prématurité: l'immaturité du sphincter inférieur de l'œsophage (SIO), l'immaturité de la motricité digestive et le retard de la vidange gastrique qu'elle entraine, le faible volume et la faible longueur de l'œsophage, les quantités de liquide plus importantes par rapport au poids du prématuré, l'alimentation parune sonde gastrique.

Le mécanisme prédominant du RGO est la relaxation transitoire du SIO [2]. Les deux principaux facteurs associés sont la diminution du péristaltisme œsophagien qui entraine une diminution de la progression du liquide régurgité vers l'estomac et la position « horizontale » permanente [3, 4] Le premier défi lors de l'évaluation d'un enfant prématuré suspect de RGO est de faire le bon diagnostic. Il est important de distinguer deux notions: RGO (symptôme) ou GER: phénomène physiologique qui survient plusieurs fois chaque jour chez des enfants en bonne santé; RGO (maladie) GERD: qui provoque des symptômes et des complications. La deuxième difficulté est liée au choix et à la hiérarchisation des examens complémentaires. Le dernier défi est de définir une stratégie thérapeutique sure [5].

## Patient et observation

Il s'agit d'un nouveau-né prématuré né à 33 semaines et 6 jours de gestation par voie basse spontanée après rupture prématurée des membranes. L'antibiothérapie maternelle a été bien conduite. La mère était âgée de 24 ans en bonne santé. Le nouveau-né est admis en unité de soins intensifs néonatals pour prématurité et risque infectieux. Il a bénéficié tout au long du séjour d'un monitoring cardiovasculaire ainsi que d'une surveillance de la saturation en oxygène. Le poids de naissance était de 2050 g, la taille: 42 cm, périmètre crânien: 31 cm et le score d´Apgar était de 8 et 9 à 1 et à 5 minutes de vie, respectivement. Après prise en charge correcte du risque infectieux, les antibiotiques ont étaient arrêtés à 48h de vie devant un bilan infectieux négatif. Il a été alimenté au lait maternel en utilisant une sonde nasogastrique les premiers jours, puis l´alimentation orale a été introduite progressivement. Le bébé était sous caféine par voie orale en prévention du risque d'apnée: 20mg/kg en dose de charge suivi de doses d´entretien de 5 mg/kg/j. Le premier jour il a présenté des épisodes d'apnées ayant nécessités un traitement par la stimulation et l´augmentation transitoire de la FIO2 sous lunettes à haut débit. Les épisodes d'apnées, initialement résolus par ce traitement, étaient de plus en plus fréquents vers le 7^ème^ jour et de plus en plus profonds au fur et à mesure que la ration per os de lait augmentait. Les examens complémentaires réalisés pour rechercher une cause possible de ces apnées: échographie crânienne, glycémie, ionogramme sanguin et urinaire, C-réactive protéine, examen des urines et hémoculture étaient normaux. L'électroencéphalogramme a été réalisé pour écarter toute anomalie neurologique et était normal. Les analyses pour le dépistage des erreurs innées du métabolisme étaient négatives. Devant l'apnée réfractaire, le traitement par Gaviscon (Alginate de Sodium) était commencé le huitième jour. Les épisodes d'apnées étaient de plus associés à des bradycardies et vers le 16ème jour de vie nous avons associés au traitement un IPP inhibiteur de la pompe à proton (Inexium). L'enfant est sorti à domicile le 21^ème^ jour sans qu'aucun problème ne se reproduise. Sur la base de l´examen physique, des analyses de laboratoire et des examens complémentaires nous avons retenu le diagnostic de reflux gastro œsophagien compliqué d'épisodes d´apnée et de bradycardie. Le nouveau-né a été revu 2 semaines plus tard en bon état.

## Discussion

Le reflux gastro-œsophagien est souvent suspecté dans diverses manifestations cliniques survenant chez le prématuré. Dhillon AS et al [1], au terme d'une enquête menée dans 77 services de réanimation néonatale, sur les critères du diagnostic du RGO retrouvaient les résultats suivants: vomissements, intolérance alimentaire et régurgitations dans 71% des cas; apnées (69% des cas); bradycardies (48%); désaturations (31%); présence de lait en oropharyngé (23%); difficultés respiratoires et inhalation (19%); inconfort, irritabilité (15%); faible prise pondérale (6%) et une toux (4%). Plusieurs études retrouvent un lien entre ces différents symptômes et le RGO. Ainsi pour Menon AP. et al [6] il y'a 14 fois plus d'apnées prolongées après des épisodes de reflux documentés par pHmétrie et manométrie. Une étude expérimentale a montré que l'instillation de liquides dans le larynx entraine une stimulation des chémorécepteurs et aboutit à une apnée. [7] Herbst JJ. et al [8] retrouvent un lien direct entre RGO, détresse respiratoireet apnées. Cependant ces signes cliniques ne sont pas spécifiques du RGO. Ainsi pour Di Fiore JM. et al [9], la survenue d'un épisode de RGO pendant une apnée n'augmente pas la durée de l'apnée et n'affecte pas la saturation et la fréquence cardiaque minimales. Par ailleurs Omari TI. et al rapportent qu'il n'y a pas plus de régurgitations dans le groupe symptomatique [2]. La responsabilité du reflux dans les événements pathologiques quoique très probable est donc loin d'être démontrée. Pour confirmer le diagnostic, il existe beaucoup de méthodes d'exploration du RGO mais très peu de consensus: la pHmétrie sur 24hest la technique de référence pour la détection du reflux acide dans l'œsophage avec une sensibilité et une spécificité supérieure à 90%. Elle permet de déterminer le reflux index (RI). (Reflux Index = tps avec pH<4; il est pathologique si >12% dans la première année de vie) [10, 11]. Dans notre cas la pHmétrie n'a pu être réalisée par ce qu'elle est indisponible dans notre structure.

Néanmoins c'est un examen qui a ses limites et qui peut être mis en défaut dans certains cas: RI< seuil lors d'épisodes de RGO brefs mais symptomatiques. Il détecte uniquement le RGO acide. La Valeur du pH seuil et du RI chez prématuré. Les résultats sont influencés par les modalités d'alimentation [12–14]. L' impédancemétrie œsophagienne est une technique de mesure de l'impédance intraluminale qui repose sur la détection de la variation de la pseudo-résistance (l'impédance) intraluminale œsophagienne lors du passage d'un bolus le long d'un segment de mesure déterminé. Elle peut identifier un mouvement antérograde (déglutition) ou rétrograde (reflux, éructation). Couplé à la pHmétrie, elle permet d'obtenir de meilleures mesures de la sévérité, du pronostic et de la réponse aux traitements [15]. On l'utilise chez le nourrisson principalement dans la détection du reflux gastro œsophagien. C'est une technique extrêmement sensible (bolus de 0.1 ml) dont les résultats sont reproductibles et elle n'est pas pH-dépendante. Lamanométrie œsophagienne est peu spécifique et peu sensible, peu prédictive de la réponse aux traitements médicaux ou chirurgicaux. Elle est utile pour le diagnostic de trouble de la motilité pour des enfants avec endoscopie normale ou pour déterminer la position du sphincter inférieur de l'œsophage pour placer une sonde. Le transit oesogastro duodénal est de réalisation difficile chez le prématuré. L'endoscopie et la biopsie de l'œsophage distal permettent de poser un diagnostic précis de reflux gastro œsophagien.Cependant l'endoscopie aussi a ses limites : elle a des critères subjectifs et non spécifiques (érythème muqueux, pâleur, modification schémas vasculaire) Les limites de la biopsie sont en rapport avec ses critères peu spécifiques et peu sensibles (éosinophilie, hyperplasie basilaire). Enfin, des critères histologiques absents n'éliminent pas un reflux gastro œsophagien.Nous n'avons pas pu réaliser ces examens car ils étaient soit très couteux soit indisponibles Le traitement du RGO du prématuré est très controversé. Cela est lié en partie à l'absence d'études randomisées et au manque d'évaluation de la majorité des mesures appliquées. Ce traitement anti reflux est instauré si les apnées sont fréquents, prolongés ou associée à des bradycardies et ou des désaturation. La prise en charge associe des mesures générales qui réduisent le risque d´apnée, un support respiratoire adéquat et l'utilisation de la caféine. Dans la plupart des centres, les mesures non médicamenteuses sont de plus en plus favorisées. Elles apparaissent comme le seul traitement à avoir démontré son efficacité quand les enfants sont posturés en proclive ou en décubitus latéral gauche. Dans notre cas elle a permis une légère amélioration des symptômes. Chez notre patient nous n'avons pas pu utiliser d'épaississants car notre volonté était de privilégier le lait maternel. Malgré une large utilisation, il existe peu de données dans la littérature chez l'enfant prématuré. Et les études chez le nourrisson et le jeune enfant montrent des résultats contradictoires. Si les études montrent une nette diminution du nombre de régurgitations, il n'y a en revanche pas de bénéfice démontré en termes de diminution du contact acide avec la muqueuse œsophagienne et ses complications en particulier broncho-pulmonaires [15, 16]. Nous n'avons pas retrouvés d'études évaluant l'efficacité des antiacides chez l'enfant prématuré. Ce qui fait que nous ne les avons pas utilisés chez notre patient.Les études chez le nourrisson et le grand enfant ont des résultats contradictoires. Certaines études montrent une réduction du RI sur la pHmétrie [17]. Pour d'autres il n y a pas d'effet clinique [18]. Les études sur l'effet des prokinétiquessur les RGO du prématuré sont rares. Mais des effets toxiques sur le rythme cardiaque ont été démontrés pour tous les prokinétiques (cisapride, dompéridone, métoclopramide) chez l'enfant plus grand [19]. Il y a peu de données sur l'indication des inhibiteurs de la pompe à protons dans les RGO du prématuré. Mais dans notre cas il semble que leur introduction associé à l'augmentation de l'âge de l'enfant et au traitement postural aient joué un rôle important dans le traitement du RGO et de ses symptômes. Des auteurs retrouvent une baisse de l'acidité gastrique, une diminution du nombre et de la durée des épisodes de reflux acide mais sans réduction de la symptomatologie.

## Conclusion

Le RGO du prématuré est une réalité physiologique et parfois une réalité pathologique. Ses signes sont peu spécifiques et son diagnostic est difficile à confirmer. Malgré tout son traitement ne doit pas être banalisé mais il ne faut pas céder à la tentation de l'escalade thérapeutique. Il est un besoin urgent d'études collaboratives entre néonatologistes et gastropédiatres pour évaluer les problèmes liés au reflux chez le prématuré et proposer une stratégie thérapeutique sure [20]. Il est nécessaire de réaliser des études randomisées contrôlées pour évaluer les différents traitements proposés contre le reflux gastro œsophagien symptomatique du nouveau-né prématuré.

## References

[1] Dhillon AS, Ewer AK (2004). Diagnosis and management of gastro-oesophageal reflux in preterm infants in neonatal intensive care units. Acta Paediatr..

[2] Omari TI, Barnett CP, Benninga MA, Lontis R, Goodchild L, Haslam RR, Dent J, Davidson GP (2002). Mechanisms of gastro-oesophageal reflux in preterm and term infants with reflux disease. Gut..

[3] Ewer AK, James ME, Tobin JM (1999). Prone and left lateral positioning reduce gastro-oesophageal reflux in preterm infants. Arch Dis Child Fetal Neonatal Ed..

[4] Omari TI, Rommel N, Staunton E, Lontis R, Goodchild L, Haslam RR, Dent J, Davidson GP J (2004). Paradoxical impact of body positioning on gastroesophageal reflux and gastric emptying in the premature neonate. J Pediatr..

[5] Clark RH, Spitzer AR (2009). Patience is a virtue in the management of gastroesophageal reflux. J Pediatr..

[6] Menon AP, Schefft GL, Thach BT (1985). Apnea associated with regurgitation in infants. J Pediatr..

[7] Davies AM, Koenig JS, Thach BT (1988). Upper airway chemoreflex responses to saline and water in preterm infants. J Appl Physiol (1985)..

[8] Herbst JJ, Minton SD, Book LS (1979). Gastroesophageal reflux causing respiratory distress and apnea in newborn infants. J Pediatr..

[9] Di Fiore JM, Arko M, Whitehouse M, Kimball A, Martin RJ (2005). Apnea is not prolonged by acid gastroesophageal reflux in preterm infants. Pediatrics..

[10] (1992). A standardized protocol for the methodology of esophageal pH monitoring and interpretation of the data for the diagnosis of gastroesophageal reflux. Working Group of the European Society of Pediatric Gastroenterology and Nutrition. J Pediatr Gastroenterol Nutr..

[11] Colletti RB, Christie DL, Orenstein SR (1995). Statement of the North American Society for Pediatric Gastroenterology and Nutrition (NASPGN): indications for pediatric esophageal pH monitoring. J Pediatr Gastroenterol Nutr..

[12] Mitchell DJ, McClure BG, Tubman TR (2001). Simultaneous monitoring of gastric and oesophageal pH reveals limitations of conventional oesophageal pH monitoring in milk fed infants. Arch Dis Child..

[13] Grant L, Cochran D (2001). Can pH monitoring reliably detect gastro-oesophageal reflux in preterm infants?. Arch Dis Child Fetal Neonatal Ed..

[14] Omari TI, Davidson GP (2003). Multipoint measurement of intragastric pH in healthy preterm infants. Arch Dis Child Fetal Neonatal Ed..

[15] López-Alonso Manuel, MOYA Maria Jose, CABO Jose Antonio (2006). Twenty-four-hour esophageal impedance-pH monitoring in healthy preterm neonates: rate and characteristics of acid, weakly acidic, and weakly alkaline gastroesophageal reflux. Pediatrics..

[16] Vandenplas Y, Hachimi-Idrissi S, Casteels A, Mahler T, Loeb H (1994). A clinical trial with an “anti-regurgitation” formula. Eur J Pediatr..

[17] Aggett PJ, Agostoni C, Goulet O, Hernell O (2002). Antireflux or antiregurgitation milk products for infants and young children: a commentary by the ESPGHAN Committee on Nutrition. J Pediatr Gastroenterol Nutr..

[18] Buts JP, Barudi C, Otte JB (1987). Double-blind controlled study on the efficacy of sodium alginate (Gaviscon) in reducing gastroesophageal reflux assessed by 24 h continuous pH monitoring in infants and children. Eur J Pediatr..

[19] Omari TI, Haslam RR, Lundborg P, Davidson GP (2007). Effect of omeprazole on acid gastroesophageal reflux and gastric acidity in preterm infants with pathological acid reflux. J Pediatr Gastroenterol Nutr..

[20] Mouterde O, Ferreti E (2008). Réflexions sur le reflux gastro-oesophagien du prématuré. Medecine & Enfance..

